# Inflammation shapes pathogenesis of murine arrhythmogenic cardiomyopathy

**DOI:** 10.1007/s00395-020-0803-5

**Published:** 2020-06-12

**Authors:** Nadine Lubos, Svenja van der Gaag, Muhammed Gerçek, Sebastian Kant, Rudolf E. Leube, Claudia A. Krusche

**Affiliations:** Institute of Molecular and Cellular Anatomy, RWTH Aachen University, 52074 Aachen, Germany

**Keywords:** Cardiomyopathy, Inflammation, Immune cells, Desmosome, Desmoglein, Chronic disease progression

## Abstract

Arrhythmogenic cardiomyopathy (AC) is an incurable genetic disease, whose pathogenesis is poorly understood. AC is characterized by arrhythmia, fibrosis, and cardiodilation that may lead to sudden cardiac death or heart failure. To elucidate AC pathogenesis and to design possible treatment strategies of AC, multiple murine models have been established. Among them, mice carrying desmoglein 2 mutations are particularly valuable given the identification of desmoglein 2 mutations in human AC and the detection of desmoglein 2 auto-antibodies in AC patients. Using two mouse strains producing either a mutant desmoglein 2 or lacking desmoglein 2 in cardiomyocytes, we test the hypothesis that inflammation is a major component of disease pathogenesis. We show that multifocal cardiomyocyte necrosis initiates a neutrophil-dominated inflammatory response, which also involves macrophages and T cells. Increased expression of *Ccl2*/*Ccr2*, *Ccl3*/*Ccr5,* and *Cxcl5*/*Cxcr2* mRNA reflects the observed immune cell recruitment. During the ensuing acute disease phase, *Mmp12*^+^ and *Spp1*^+^ macrophages and T cells accumulate in scars, which mature from cell- to collagen-rich. The expression of *Cx3cl1*/*Cx3cr1*, *Ccl2*/*Ccr2,* and *Cxcl10*/*Cxcr3* dominates this disease phase. We furthermore find that during chronic disease progression macrophages and T cells persist within mature scars and are present in expanding interstitial fibrosis. *Ccl12* and *Cx3cl1* are predominant chemokines in this disease phase. Together, our observations provide strong evidence that specific immune cell populations and chemokine expression profiles modulate inflammatory and repair processes throughout AC progression.

**Electronic supplementary material:**

The online version of this article (10.1007/s00395-020-0803-5) contains supplementary material, which is available to authorized users.

## Introduction

Arrhythmogenic cardiomyopathy (AC), previously referred to as arrhythmogenic right ventricular cardiomyopathy (ARVC), is a genetic disease that is characterized by arrhythmia and cardiac dilation. It is complicated by sudden cardiac arrest and leads to heart failure [10, 25]. A hallmark feature of late disease stages is the presence of excessive fibrofatty tissue within both ventricular walls [20]. The human disease can be separated into (i) a concealed preclinical phase with a risk of life-threatening arrhythmia, (ii) a phase with the onset of structural abnormalities and overt electrical disorders, and (iii) a chronic phase of progressive heart failure [62]. The pathogenic pathways leading to morphological disease onset, acute fibrotic scar formation and chronic deterioration of heart structure and function are still poorly understood. Mechanical dysfunction and complex tissue responses are discussed as factors determining the disease process [6, 8, 19, 39, 44]. Besides symptomatic treatment, a causal therapy is not available to date [64].

AC has been linked to mutations in genes encoding proteins of desmosomal cell–cell adhesions [29]. Among them, mutations of the desmosomal cadherin desmoglein 2 gene (denoted as *Dsg2*) have obtained particular attention given their severe phenotype [34] and the consistent and specific detection of desmoglein 2-autoantibodies in AC patients and AC animal models [13].

To better understand the pathomechanisms of AC and to explore possible therapies, multiple murine mouse models have been established [51]. To date, several mouse strains have been described that either overexpress mutant desmoglein 2 protein (denoted as DSG2) [52], lack DSG2 in cardiomyocytes [38] or constitutively produce mutant DSG2 [14, 42] reflecting situations encountered in human AC patients ranging between homo- and heterozygosity and between mutant and absent DSG2 [17, 18, 58]. An AC-like phenotype was reported in all transgenic mouse lines presenting with cardiomyocyte death, inflammation, fibrosis, and cardiac dysfunction [40, 42, 52]. Morphological disease onset with localized lesion formation was observed 2 weeks after birth. It is followed by the acute disease phase during which lesions are transformed into mature fibrous scars by the age of 10–12 weeks. At this time, the chronic disease phase sets in with progressive cardiac wall alterations in aging mice [31, 40].

It is has been reported that inflammation is a component of AC disease initiation and progression with multiple reports on the detection of immune cells in various disease stages [5, 8, 11, 15, 25, 39, 40, 59, 60]. A systematic study of the sequential responses in relation to the different disease stages and a thorough characterization of the disease onset, however, is still lacking. The goal of the current study was therefore to determine whether and how inflammation contributes to the different disease stages of murine AC and to dissect the cellular responses involved. To do this, special emphasis was on the examination of local responses by immunohistochemistry to capture the specific cell types and on the production of inflammatory cytokines and their receptors to elucidate mechanisms determining very early alterations and to connect the consecutive pathologies occurring during scar formation and the chronic phase.

## Materials and methods

### Animals and tissue sampling

Two different *Dsg2* mutant mouse strains were used in this study. Homozygous *Dsg2*^*MT*^ mice carry *Dsg2* alleles lacking exons 4, 5, and 6 and encoding a truncated DSG2 protein of approximately 110 kDa which lacks parts of the extracellular domains 1 and 2 [42]. The mutant DSG2 still localizes to the intercalated discs but is less abundant than the wild-type protein [41, 42]. Additionally, *Dsg2*^*MT*^ mice present a pronounced reduction of desmosomes at the intercalated discs [39, 40]. Approximately two-thirds of the *Dsg2*^*MT*^ mice die during embryogenesis. Surviving *Dsg2*^*MT*^ individuals are born apparently healthy and develop myocardial lesions around day 14 after birth [40, 42].

Cardiomyocyte-specific desmoglein 2 knock-out (*Dsg2*^*cKO*^) mice are homozygous carriers of a *Dsg2* allele containing two loxP sites, which flank exons 4 and 5 of the *Dsg2* gene (*Dsg2*^*flox(E4−5)*^; [39]). *Dsg2*^*flox(E4−5)*^ mice were then crossed with B6.FVB-Tg(Myh6-cre)2182Mds/J mice containing a *Myh6-Cre* transgene that is specifically expressed in cardiomyocytes after E10 [1]. The Cre recombinase-mediated excision of exons 4 and 5 leads to the mutant allele *Dsg2*^cKO^ encoding a nonfunctional aminoterminal DSG2 polypeptide (for further details see [39]). *Dsg2*^*cKO*^ mice show much less embryonic lethality than *Dsg2*^*MT*^ mice probably because some wild-type DSG2 protein is still present during midgestation. After birth, however, DSG2 is no longer detectable in the myocardium of *Dsg2*^*cKO*^ mice [39]. *Dsg2*^*cKO*^ mice develop myocardial lesions slightly later than *Dsg2*^*MT*^ mice around day 18. Besides these differences, the cardiac phenotype is the same for *Dsg2*^*MT*^ and *Dsg2*^*cKO*^ mice from 4 weeks onwards as determined by histomorphology, intercalated disc ultrastructure, electrocardiography, Cx43 distribution, and CD45 immunoreactivity [39]. Neither heterozygous *Dsg2* mutant (*Dsg2*^*HT*^) mice nor homozygous *Dsg2*^*flox(E4−5)*^ mice display a cardiac phenotype.

Mice were housed in the animal facility of the University Hospital of RWTH Aachen University. They received a standard rodent lab diet (Ssniff, Soest, Germany) and had free access to food and water. The experiments were conducted in accordance with the guidelines for the care and use of laboratory animals and were approved by the Ministry for Environment, Agriculture, Conservation and Consumer Protection of the State of North Rhine-Westphalia (reference number 84-02.04.2015.A190 and A4 notifications for killing animals for scientific purposes).

Animals were killed by cervical dislocation. The thoracic cavity was opened after cervical dislocation and gross heart morphology was documented in situ. The heart was then removed and dissected in two different ways for the ensuing analyses:

i. Hearts were divided into an apical and a basal part along the transverse plane so that both halves contained diseased myocardium. The tip of the heart was used for RNA isolation by homogenization in RNA isolation buffer (PeqLab Gold RNA isolation kit; VWR, Darmstadt, Germany). The homogenate was stored at − 80 °C until further processing. The remaining basal part of the heart was either chemically fixed in formaldehyde or cryofixed in liquid nitrogen.
ii. To obtain quantitative data on mRNA expression of the left and the right ventricular wall, the atria were first cut off. The right ventricular free wall was then removed, cleaned from adherent blood, and homogenized in RNA isolation buffer. The septum was inspected for fibrotic scar tissue or structural abnormalities during dissection. The remaining left ventricle was opened, blood clots were removed and the left ventricular myocardium was homogenized in RNA isolation buffer. All homogenates were stored at − 80 °C until RNA isolation.

### RNA isolation, cDNA synthesis, and qRT-PCR

Total RNA isolation, cDNA synthesis, and qRT-PCR experiments were performed as described [31]. In brief, total RNA was isolated using the PeqGOLD Total RNA Kit. 1 µg of RNA was reverse transcribed using the Transcriptor First Strand kit (Roche, Mannheim, Germany) utilizing oligo dT-primer. The qRT-PCRs were performed with the help of the Light Cycler Taqman Master Kit, Universal Probe Library (UPL) probes (both Roche) and the primer pairs listed in Table 1.

**Table 1:** Real-time PCR primer pairs and corresponding UPL probes

Gene	ID	Forward primer	Reverse primer	UPL
*Ccl7*	NM_013654.3	aggatctctgccacgcttc	ttgacatagcagcatgtggat	#40
*Ccl12*	NM_011331.2	gtccggaagctgaagagcta	tctccttatccagtatggtcctg	#71
*Cxcl5*	NM_009141.2	gggaaaccattgtccctga	ccgatagtgtgacagataggaaag	#4
*Cxcl10*	NM_021274.1	gctgccgtcattttctgc	tctcactggcccgtcatc	#3
*Cx3cl1*	NM_009142.3	cacccagaagccagtgactc	cctcactctcaggagccaac	#31
*Ccr3*	NM_009914.4	gagcatcaacaacacgttcc	tgaaagtgtgatcttgggacaa	#77
*Cxcr2*	NM_009909.3	accctctttaaggcccacat	aggacgacagcgaagatgac	#29
*Cxcr3*	NM_009910.2	gcgtgtactgcagctagtgg	tagcagtaggccatgaccaga	#18
*Cxcr4*	NM_009911.3	gtctatgtgggcgtctggat	acgtcggcaaagatgaagtc	#63
*Cx3cr1*	NM_009987.3	ccatctgctcaggacctcac	caaaattctctagatccagttcagg	#10
*Lgals 3*	NM_001145953	gcctaccccagtgctcct	ggtcatagggcaccgtca	#18
*Il-10*	BC120612.1	cagagccacatgctcctaga	tgtccagctggtcctttgtt	#41
*Tnf α*	BC137720.1	ctgtagcccacgtcgtagc	ttgagatccatgccgttg	#25
*Spp1*	AF515708.1	cccggtgaaagtgactgatt	ttcttcagaggacacagcattc	#82
*Il-1β*	NM_008361.3	agttgacggaccccaaaag	gaagctggatgctctcatca	#26
*Ccl2*	NM_011333.3	caggtccctgtcatgcttct	gtggggcgttaactgcat	#40
*Ccl3*	NM_011337.2	cccagccaggtgtcattt	ctgcctccaagactctcagg	#85
*Ccr2*	NM_009915.2	acctgtaaatgccatgcaagt	tgtcttccatttcctttgatttg	#27
*Ccr5*	NM_009917.5	aatatagcgttcttggattaagtgg	acggctaaaaatactttcaaggaa	#9
*Cd45*	BC167212.1	cgggatgagacagttgatga	gtattctgcgcacttgttcct	#88
*Ym1*	BC061154.1	aagaacactgagctaaaaactctcct	gagaccatggcactgaacg	#88
*Cd3e*	NM_007648.4	ccagagggcaaaacaagg	gcgatgtctctcctatctgtca	#49
*Cd68*	NM_009853.1	gctgttcaccttgacctgct	tcacggttgcaagagaaaca	#27
*F4/80*	BC075688.1	ggaggacttctccaagcctatt	aggcctctcagacttctgctt	#42
*HMBS*	NM_013551.2	aagttcccccacctggaa	gacgatggcactgaattcct	#42

### Histology and immunohistochemistry

For paraffin embedding, dissected hearts were first fixed overnight by submersion in 4% neutrally buffered formaldehyde in phosphate-buffered saline (PBS). After rinsing in PBS for 1 h samples were dehydrated in an ascending isopropanol series (50%, 70%, 90%, 100%). After 1 h in 100% isopropanol at 60 °C, tissues were transferred to liquid paraffin (60 °C). After 2 h paraffin was exchanged and after overnight incubation tissues were embedded in paraffin blocks.

For immunohistochemical staining of fresh frozen samples hearts were cut into two halves along the transverse plane. The cut surface was placed on the bottom of a TissueTek cryomold and the tissue was then carefully covered with TissueTek OCT compound (Science Services, Munich, Germany). Tissue was shock frozen in liquid nitrogen and stored at − 40 °C until sectioning.

To assess the histology of mutant and healthy hearts 5 µm thick serial paraffin sections were prepared and stained with hematoxylin-eosin (HE), AZAN trichrome and von Kossa stain as described before [39, 40, 42].

For immunohistochemical staining of the CD44, CD3, CD45R, and myeloperoxidase (MPO), 5 µm thick paraffin sections were used. After deparaffinization including peroxidase blocking with 3% H_2_O_2_ in 70% ethanol sections were rehydrated in PBS. Prior to the incubation with the primary antibodies specific antigen retrieval was performed following different antigen-dependent regimen: (i) CD44 and TNC: 40 min incubation in 10 mM/L citrate buffer pH 6 at 94 °C followed by 20 min cool down to room temperature; (ii) CD3: 40 min incubation in boiling 10 mM/L citrate buffer pH 6; (iii) CD45R: 30 min incubation in 50 mM EDTA buffer at room temperature to remove calcium; (iv) MPO: 2 × 10 min boiling in 10 mM/L citrate buffer pH 6 followed by 20 min cool down to room temperature. The sections were then incubated with the first antibodies. Dilution and incubation times are provided in Table 2.

**Table 2:** List of primary antibodies

Antigen	Species/antibody type	Supplier	Catalogue number	Dilution/incubation time and temperature
Antibodies used on paraffin sections				
CD44	Rat/IgG2b monoclonal	BD Pharmingen	550,538	1:50/1 h, RT
CD3	Rabbit/IgG monoclonal	ZytomedSystems	M3070	1:100/16 h, 4 °C
MPO	Rabbit/Polyclonal	Thermo Scientific	Rb 373 A	1:500/16 h, 4 °C
CD45R	Rat/IgG2a monoclonal	eBioscience	14–0452	1:100/2 h, RT
TNC	Rabbit/Polyclonal	Faissner lab		1:10.000/1 h, RT
Antibodies used on croystat sections				
F4/80	Rat/IgG2b monoclonal	Abcam	Ab6640	1:100/1 h, RT
CD3e	Armenian hamster/IgG	eBioscience	16–0031	1:100/1 h, RT
CD4	Rat/IgG2a monoclonal	eBioscience	14–0042-81	1:200/1 h, RT
CD8a	Rat/IgG2a monoclonal	eBioscience	16–0081-81	1:200/1 h, RT
CD11b	Rat/IgG2b monoclonal	eBioscience	14–0112	1:200/1 h, RT
CD11c	Armenian hamster/IgG	eBioscience	14–0114	1:100/1 h, RT
CD206	Rat/IgG2a monoclonal	BioRad	MCA2235T	1:400/1 h, RT

*RT* room temperature

The presence and localization of monocyte/macrophage and T cell subsets were assessed on 10 µm thick cryostat section using antibodies against the CD11b, CD11c, CD4, CD8, F4/80 and CD206 antigens. After cutting, sections were allowed to adhere to the slides for 30 min at room temperature. Thereafter, sections were fixed for 10 min at − 20 °C in acetone that had been precooled to − 20 °C. After rehydration in PBS, the endogenous peroxidase was blocked by treating sections with 0.3% H_2_O_2_ in PBS for 10 min in the dark. After two washing steps in PBS (5 min each) the first antibody, which was either diluted in PBS or PBS containing 1.5% BSA, was applied. Details on dilution and incubation times are given in Table 2.

After incubation with the first antibodies, paraffin and cryostat sections were washed three times for 5 min with PBS. Thereafter antibody detection was accomplished by anti-rabbit and anti-rat polymer kits or in case of the CD11c and CD3e antibody by a goat anti-hamster IgG horseradish peroxidase conjugate (see Table 3 for incubation times and dilutions).

**Table 3:** List of immunohistochemistry reagents and kits

Detection kits/antibody conjugates	Supplier	Catalogue number	Dilution / incubation time
Anti-mouse/anti-rabbit HRP Polymer System	Zytomed Systems	790-HRP	Ready to use/30 min
Simple Stain Mouse MAX PO (anti-rat)	Nichirei Biosciences Inc	414321F	Ready to use/30 min
Goat anti-Armenian hamster—HRP-conjugate	Jackson/Dianova	127-035-160	1:400/45 min
DAB Substrate Kit	Zytomed Systems	TA-060-QHDX	Visual control: 2–10 min

Two kinds of negative control experiments were performed by either omitting the first antibody or replacing the first antibody by a non-IgG control antibody of the same isotype and concentration. Spleen was used as a positive control.

### Immunohistochemical intensity score

To compare and to quantify the density of CD11b-, CD11c-, CD3-, CD4- and F4/80-positive cells within myocardial scars of *Dsg2*^*MT*^ mice, a semiquantitative score (0–4) was established (see Supplementary Fig. 1). Individual scars were scored and the mean value was calculated for each animal.

### In situ hybridization

We used the type 1 probe sets for mouse *Spp1* and *Mmp12* mRNA (VB1-14479-VT and VB1-14709-VT, respectively; Thermo Fisher Scientific) and the ViewRNA ISH Tissue Assay Kit (1-plex; Cat# 19931, Thermo Fisher Scientific). 5 µm-thick sections of 4% formaldehyde-fixed and paraffin-embedded cardiac tissue (for details see above) were placed on Surgipath™ X-tra Adhesive pre-cleaned micro slides (Leica Biosystems, Wetzlar, Germany). Further processing was done as detailed in the protocols provided by the manufacturer. Negative controls were stained without the hybridization probe or with a scrambled probe to *Mmp12*.

### Statistics

The mRNA expression data were analyzed using LightCycler 96 software 1.1 and are given as normalized ratio quantification (NRQ; [33]).

For comparisons of three groups (at disease onset at the age of 18–19 days in *Dsg2*^*cKO*^ or day 14 in the *Dsg2*^*MT*^ mice) the Kruskal Wallis test and selected Post hoc Dunn's comparison tests were used (*Dsg2*^*MT*^ mice: *Dsg2*^*WT*^ versus *Dsg2*^*MT*^ and *Dsg2*^*WT*^ vs *Dsg2*^*MT*^ + Ph; in *Dsg2*^*cKO*^ mice: *Dsg2*^*loxP*^versus *Dsg2*^*cKO*^ and *Dsg2*^*loxP*^ versus *Dsg2*^*cKO*^ + Ph). When comparing *Dsg2*^*MT*^ mice with *Dsg2*^*WT*^ mice or *Dsg2*^*cKO*^ mice with *Dsg2*^*loxP*^ control mice in the other age groups (4–6 weeks, 12 weeks and 30–32 weeks), the non-parametric Mann Whitney test was applied. Statistics were performed by using GraphPad Prism Version 5.01 (GraphPad Software, Inc, USA).

## Results

### Cardiomyocyte necrosis triggers an inflammatory response during the early stage of cardiomyopathy

**Fig. 1:**
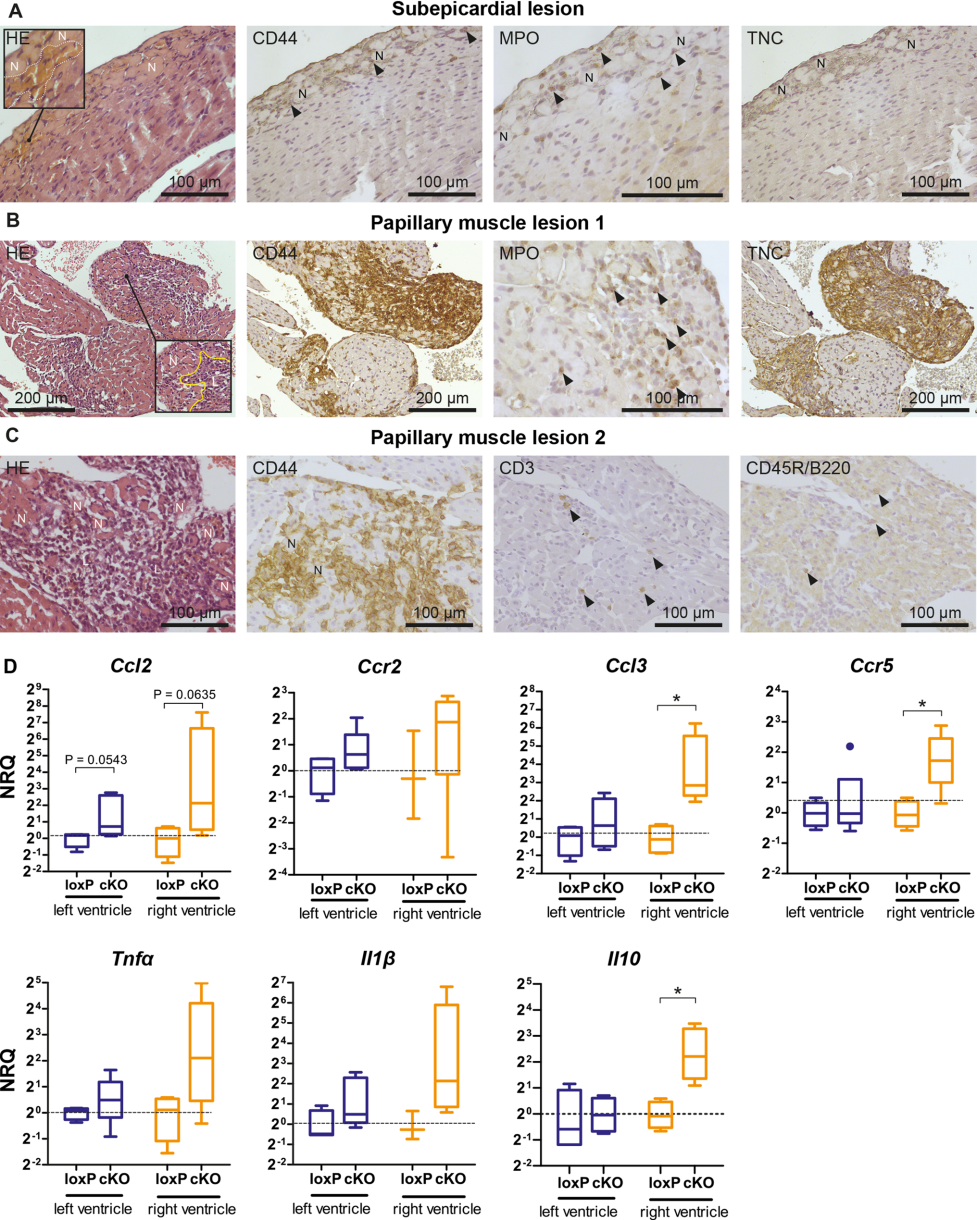
Cardiomyocyte-specific *Dsg2* knockout (*Dsg2*^*cKO*^) mice develop cardiomyocyte necrosis and an inflammatory response 18–19 days after birth. **a**–**c** The micrographs show tissue sections that were obtained from *Dsg2*^*cKO*^ mice without macroscopically detectable cardiac pathologies except for slight atrial dilation in the animal used for the sections shown in **c**. Serial sections were stained with hematoxylin–eosin (HE) or reacted with antibodies against CD44 to characterize the extent of the inflammatory infiltrate, MPO to identify neutrophil granulocytes, CD3 to detect T-lymphocytes and CD45R/B220 to identify B lymphocytes. Immunohistochemistry of the matricellular protein Tenascin C (TNC) indicates the onset of ventricular remodeling. **a** Note the presence of subepicardial necrosis (N) with adjacent enlarged blood vessels (demarcated by white broken line in the inset at left) and the detection of only very few CD44^+^ and MPO^+^ cells (arrowheads) with little to no detection of TNC indicating that an inflammatory response is just starting and that tissue remodeling has not occurred yet. **b** In contrast, the papillary muscle lesion 1 observed in the same animal presents with a strong inflammatory reaction as assessed by CD44 staining and commenced tissue remodeling (TNC positivity). The center of the lesion (L), which is delineated by a broken yellow line in the insert of the image at left is already free of cardiomyocyte remnants whereas necrotic cardiomyocytes (N) line the border of the lesion. The granular MPO staining (arrowheads) indicates the presence of neutrophil granulocytes, whereas the homogenous cytoplasmic MPO staining is indicative of macrophages. **c** The papillary muscle lesion 2 of another *Dsg2*^*cKO*^ mouse also shows an extensive inflammatory infiltrate surrounded by necrotic cardiomyocytes. A considerable number of CD3^+^ T lymphocytes and some CD45R/B220^+^ B lymphocytes are present. **d** Tukey's whisker plots of ventricle-specific mRNA detection (normalized relative quantification; NRQ) show comparisons between the expression of chemotactic and inflammatory cytokines in *Dsg2*^*cKO*^ (cKO) and *Dsg2*^*loxP*^ (loxP) control mice 18–19 days after birth. The broken line indicates the mean expression of the loxP control. None of the animals had an overt cardiac phenotype. Left ventricle: *n* = 4–5 for *Dsg2*^*cKO*^, *n* = 4 for *Dsg2*^*loxP*^; right ventricle: *n* = 4–5 for *Dsg2*^*cKO*^, *n* = 3–4 for *Dsg2*^*loxP*^. The non-parametric Mann–Whitney test was applied: *p < 0.05. Detailed statistics in Supplementary Table 1

It has been suggested that cardiomyocyte necrosis is the key pathogenic event triggering an inflammatory response in murine *Dsg2*-related AC [40, 53]. To systematically study this early response, we collected *Dsg2*^*cKO*^ hearts [39] at days 18 and 19 when the first macroscopic alterations, notably white scars and ventricular discoloration, became discernable in some but not all animals. Serial sectioning of inconspicuous hearts unveiled histological abnormalities in three out of three specimens. Figure 1a depicts a subepicardial lesion capturing an early stage of cardiomyocyte necrosis. The original size and shape of the cardiomyocytes were still maintained although the cytoplasm was amorphous and displayed strong eosinophilia. Furthermore, nuclei of the necrotic cells were either completely missing or pyknotic. Erythrocyte accumulations were seen in the vicinity of necrosis indicating enlarged capillaries or local hemorrhage (inset in Fig. 1a).

To test for the presence or absence of blood-borne immune cells, subsequent sections were reacted with anti-CD44 antibodies. Positive cells were not seen in the adjacent normal-appearing myocardium and only very few labeled cells were detected in the vicinity of necrotic cardiomyocytes. Based on their granular cytoplasmic MPO staining they could be classified as neutrophil granulocytes (Fig. 1a). CD3^+^ T cells were not enriched in this lesion and tenascin C (TNC), a sensitive indicator of tissue remodeling [37], was not expressed. Taken together and based on observations in murine myocardial infarction models [16], the depicted lesion and similar lesions can be classified as early nascent lesions.

In the same *Dsg2*^*cKO*^ heart as well as in the other *Dsg2*^*cKO*^ hearts without macroscopically visible alterations prominent changes such as myocardial necrosis and inflammation were noted in papillary muscles (Fig. 1b, c). The pathogenesis was obviously further progressed: large accumulations of inflammatory, i.e. CD44^+^ cells, had infiltrated the necrotic myocardium. The centers of the lesions were free of cardiomyocyte remnants, but necrotic cardiomyocytes lined the borders of the lesions. Homogenous cytoplasmic and granular MPO staining identified abundant macrophages and neutrophil granulocytes, respectively, in the infiltrates. Furthermore, a considerable number of CD3^+^ T cells and a few CD45R^+^ B cells were also detected. The observed removal of necrotic cardiomyocytes and the detection of immune cells belonging to the adaptive immune system support the conclusion that the papillary lesions shown in Fig. 1b, c represent stages subsequent to that shown in Fig. 1a.

**Fig. 2:**
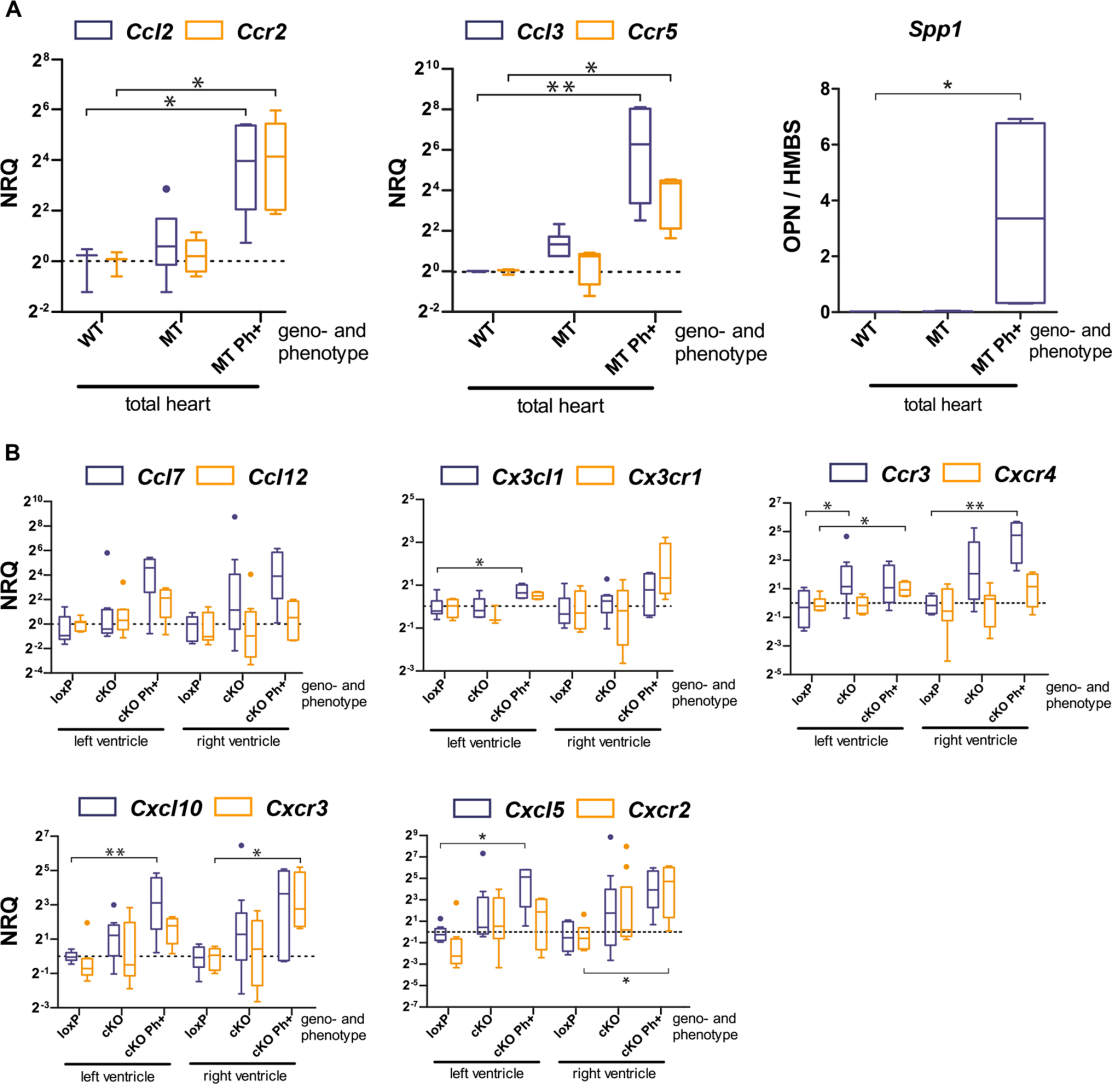
Chemokine and chemokine receptor mRNA expression increase at the onset of structural disease in the heart of *Dsg2*-mutant mice. Data are presented as Tukey's whisker plots as normalized relative quantification (NRQ). The broken line indicates the mean expression of the wild-type (WT) or *Dsg2*^*loxp*^ (loxP) control. **a** mRNA expression (NRQ) of the classical chemokines *Ccl2* and *Ccl3* and their respective receptors *Ccr2* and *Ccr5* is shown for total hearts of 2 week-old *Dsg2*^*WT*^ mice (WT; *n* = 3), *Dsg2*^*MT*^ mice without visible cardiac lesions (MT; *n* = 6) and *Dsg2*^*MT*^ mice with the overt phenotype (MT Ph + ; *n* = 5). At right the mRNA expression of the cytokine osteopontin (*Spp1*; WT: *n* = 3; MT: *n* = 3 and MT Ph + : *n* = 4) is depicted. The Kruskal Wallis test and selected post hoc Dunn's comparison tests were applied for WT versus MT and WT versus MT Ph + . *p < 0.05, **p < 0.01. Detailed statistics in Supplementary Table 2. **b** The plots show ventricle-specific mRNA expression of chemokines and chemokine receptors that are involved in the recruitment of monocytes/macrophages (*Ccl7*, *Ccl12*, *Cx3cl1*, *Cx3cr1*), T cells (*Ccr3*, *Cxcl10*, *Cxcr3*), neutrophils (*Cxcl5*, *Cxcr2*) and bone marrow-derived immune cells (*Cxcr4*) in the hearts of 18–19 day-old *Dsg2*^*cKO*^ mice either without the overt disease (cKO; *n* = 9–10) or with the overt disease (cKO Ph + ; *n* = 4) and *Dsg2*^*loxP*^ controls (loxP; *n* = 8–9). The Kruskal Wallis test and selected post hoc Dunn`s comparison tests were applied for loxP versus cKO and loxP versus cKO Ph + . *p < 0.05, **p < 0.01. Detailed statistics in Supplementary Tables 3 and 4

To further characterize the early inflammatory response, we assessed the mRNA expression of the chemokines *Ccl2*, *Ccl3,* and their respective receptors *Ccr2* and *Ccr5* that are known to be involved in the recruitment of mononuclear cells in ischemic myocardium [23, 65]. Significant upregulation was observed for *Ccl3* and *Ccr5* in the right but not the left ventricle of 18–19 day-old *Dsg2*^*cKO*^ hearts without an overt phenotype. In addition, a trend to elevated *Ccl2* mRNA expression was noted in the right ventricles. The mRNA expression of *Ccl2*, *Ccl3*, *Ccr2,* and *Ccr5* was also studied in the hearts of a second model of murine AC, i. e. in *Dsg2*^*MT*^ mice, which develop a very similar phenotype (Fig. 2a; [42]). In *Dsg2*^*MT*^ mice, disease onset occurred even earlier, namely 14 days after birth. We found that *Ccl3* mRNA was already elevated in *Dsg2*^*MT*^ hearts without macroscopically visible lesions. In hearts with visible surface scars *Ccl2*, *Ccl3*, *Ccr2,* and *Ccr5* mRNAs were all upregulated. Together, our findings suggest that *Ccl3* is one of the first upregulated cytokines during the onset of murine AC pathogenesis.

The mRNA expression of the inflammation-associated cytokines *Tnfα* and interleukin *Il1β* was highly variable in inconspicuous *Dsg2*^*cKO*^ hearts and was therefore statistically not different from that in *Dsg2*^*loxP*^ controls (Fig. 1d). Very minor differences in lesion age and size may account for the observed variability in *Tnf*α and *Il1β* mRNA production as suggested by the reported rapid changes in cytokine gene expression in ischemic murine hearts [23]. Most interestingly, anti-inflammatory cytokine *Il10* mRNA expression was significantly upregulated in the right ventricles of *Dsg2*^*cKO*^ mice.

The mRNA expression of chemokines and chemokine receptors orchestrating the recruitment of specialized immune cell subpopulations that are involved in the resolution of necrotic cells and the induction of tissue repair were then assessed in isolated left and right ventricles of *Dsg2*^*cKO*^ mice without and with overt lesions at 18–19 days (Fig. 2b). The examined chemokines and corresponding chemokine receptors were selected based on studies in myocardial infarction [2, 30, 43, 50]. Consistent with early neutrophil recruitment was the detection of significant *Cxcl5* mRNA upregulation in both ventricles and the detection of *Cxcr2* mRNA in the right ventricles of phenotypic *Dsg2*^*cKO*^ mice. Furthermore, the upregulation of *Cxcl10* and *Cxcr3* mRNA in both ventricles was taken as an indication of a beginning T cell response. Elevated *Ccl7* mRNA expression in ventricles of *Dsg2*^*cKO*^ mice in conjunction with increased *Ccl2* and *Ccr2 * in *Dsg2*^*MT*^ mice (see Fig. 2a) indicated the recruitment of CCR2^+^ inflammatory monocytes into the mutant heart that likely differentiate into macrophages. In sum**,** the chemokine profile correlated well with the composition of the immune cell infiltrate found during disease onset (Fig. 1b, c).

### Inflammation is involved in the formation of purely fibrotic and calcifying scars

**Fig. 3:**
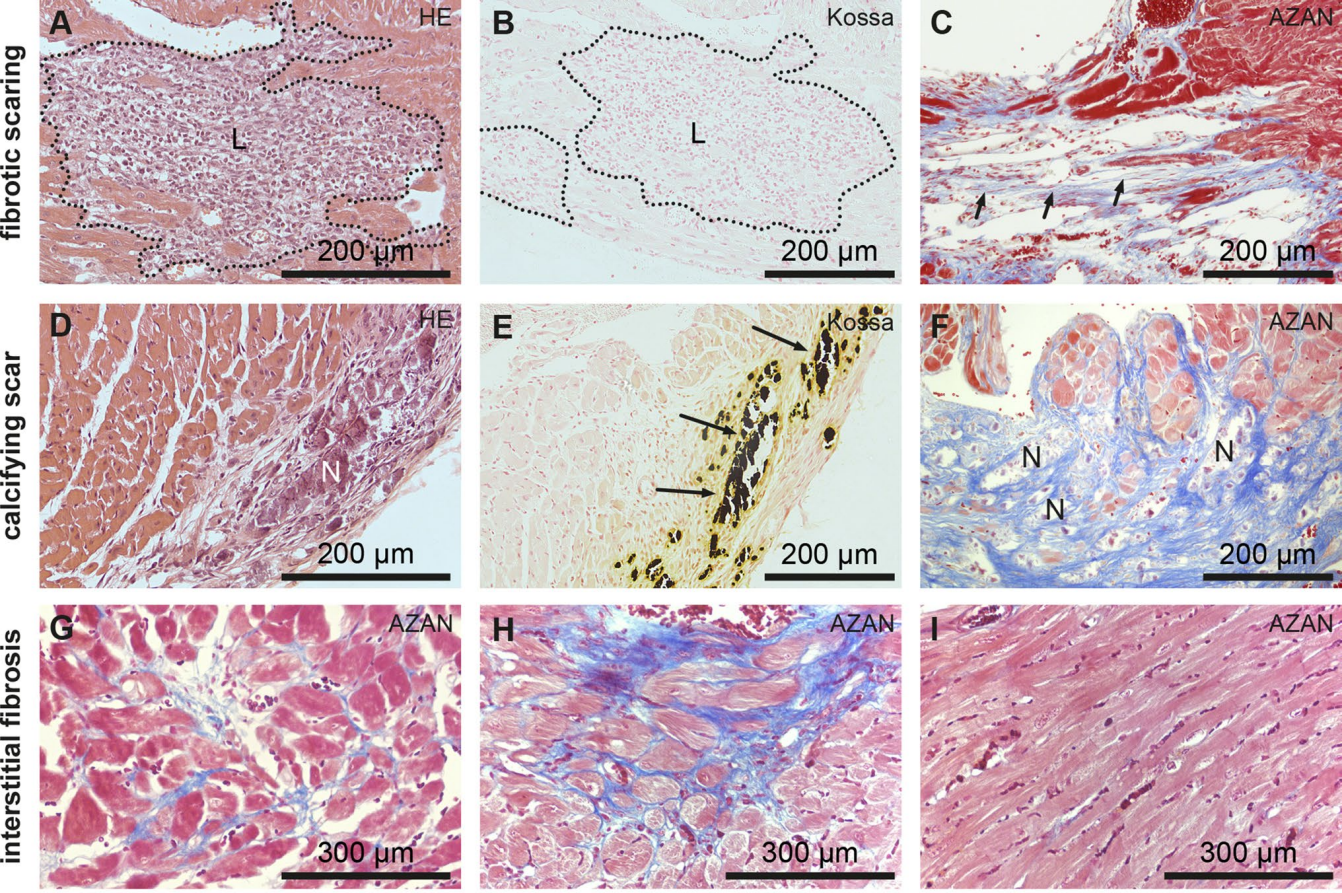
Purely fibrotic or calcifying scars form during the acute disease phase in response to extensive cardiomyocyte necrosis at disease onset while interstitial fibrosis indicates ongoing myocardial remodeling during the chronic disease phase in *Dsg2*^*MT*^ and *Dsg2*^*cKO*^ mice. **a**, **b**, **d,** and **e** show immature scars of juvenile, 4 week-old *Dsg2*^*cKO*^ mice with (**a**, **b**) and (**d**, **e**) being serial sections of the same animals, respectively. **c**, **f** show micrographs of mature scars from adult, 3 month-old *Dsg2*^*MT*^ mice after AZAN trichrome staining. Calcified necrotic areas are denoted by N, black calcium deposits by arrows and fibrotic lesions by L. Note that the cardiomyocyte-free mature fibrotic lesion in **c** consists mainly of connective tissue (short arrows) that extends to the endo- and perimysium of the adjacent intact-appearing myocardium and is coupled to wall thinning, whereas the mature calcified lesion in **f** is associated with extremely collagen-rich thick connective tissue. The micrographs in **g**–**i** show sections of 30–40 week-old *Dsg2*^*MT*^ hearts during the chronic disease phase. AZAN staining reveals that besides the scars depicted in **c** and **f** increasingly areas with loose (**g**) and dense (**h**) interstitial fibrosis are present. **i** depicts myocardium that is not yet affected

Tissue remodeling occurs after cardiomyocyte necrosis and initial recruitment of inflammatory cells in *Dsg2*^*MT*^ and *Dsg2*^*cKO*^ mice invoking two types of replacement fibrosis: predominantly collagenous scars (Fig. 3a–c) and scars containing calcified necrotic cardiomyocytes (Fig. 3d–f). In collagenous scars, necrotic cardiomyocytes are completely phagocytosed by infiltrating immune cells leaving only endomysial and perimysial connective tissue. Cardiac wall thinning is typically observed in these instances. On the other hand, calcification occurs when necrotic cardiomyocytes are not phagocytosed. The calcified regions elicit fulminant adjacent fibrosis leading to local cardiac wall thickening, which manifests as solid white scars. To extend previous reports of CD45^+^ immune cells in immature scars [39, 40] and to dissect the immune cell types associated with the different fibrotic responses, we decided to study the expression of specific immune cell markers during scar formation between 4 and 12 weeks.

**Fig. 4:**
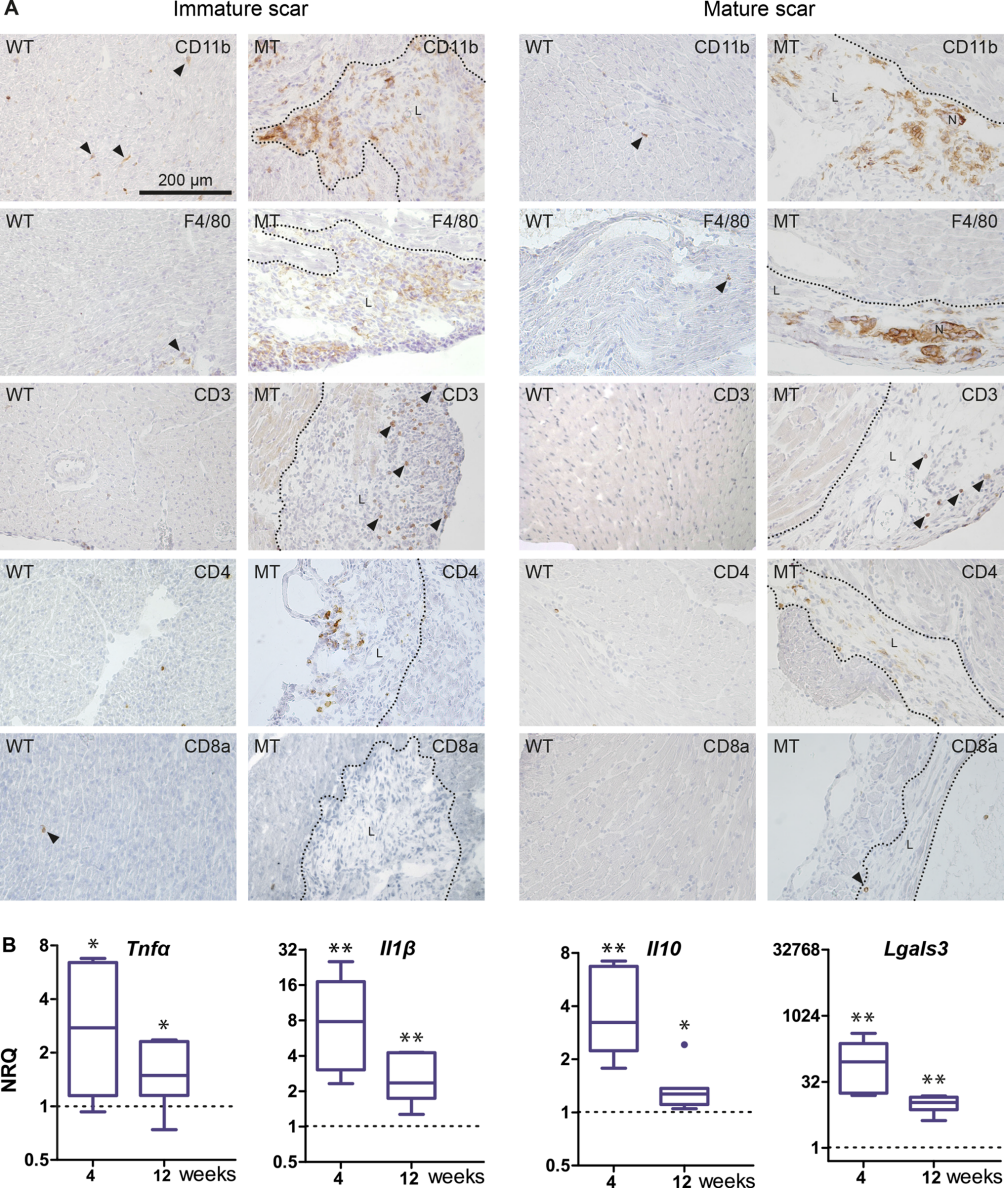
Comparison of immature and mature scars in *Dsg2*^*MT*^ mice identify macrophages as the predominant immune cell population in both and shows that increased expression of inflammation-associated cytokines is strongly reduced in mature scars. **a** The micrographs show tissue cryosections that were obtained from 4 and 12 week-old *Dsg2*^*MT*^ mice (immature scars [*n* = 3] and mature scars [*n* = 4], respectively) and corresponding wild-type *Dsg2*^*WT*^ mice (WT; *n* = 3–5) after reaction with antibodies directed against various immune cell-specific surface antigens. All images are shown at the same magnification (scale bar in the picture at the upper left corner). Note that CD11b^+^ and F4/80^+^ macrophages are the predominant immune cell type in immature and mature scars and that the number of CD3^+^ T cells is higher in immature than in mature scars. Typical round-shaped CD4^+^ T cells are present in immature scars, whereas primarily spindle-shaped CD4^+^ cells are detected in mature scars. CD8a^+^ cells were only rarely detected in *Dsg2*^*MT*^ and control hearts (arrowheads). **b** Tukey's whisker plots show the results of qRT-PCR analyses assessing the mRNA expression (normalized relative quantification; NRQ) of inflammation-associated cytokines in total hearts of *Dsg2*^*MT*^ (*n* = 5–7) and *Dsg2*^*WT*^ mice (*n* = 5–7) at 4 and 12 weeks. They are significantly upregulated in *Dsg2*^*MT*^ mice of both age groups compared to the wild-type controls (broken lines). However, their expression decreases with age. The non-parametric Mann Whitney test was applied to compare *Dsg2*^*WT*^ and *Dsg2*^*MT*^ expression within each age group. *p < 0.05 and **p < 0.01. More detailed statistic data are provided in Supplementary Table 5

The majority of infiltrating cells consisted of CD11b^+^ and F4/80^+^ macrophages (Fig. 4a). In addition and in contrast to the incipient lesions, a considerable number of CD3^+^ T cells and a few round-shaped CD4^+^ cells, which are most likely T-helper cells, were present (Fig. 4a). CD11b^+^ and F4/80^+^ macrophages also resembled the dominant immune cell type in mature, purely collagenous and calcified scars of 12 week-old *Dsg2*^*MT*^ mice (Fig. 4a). In comparison to the immature scars, fewer but still increased CD3^+^ and CD4^+^ cells were present. Only some of the CD4^+^ cells, however, fit the typical round-shaped T-helper cell phenotype whereas the majority was spindle-shaped most likely being dendritic cells (Fig. 4a) [63]. CD8a^+^ cells were detected only rarely in cardiac tissue sections without positional preference for scars and at a comparable frequency to wild-type controls (Fig. 4a). Of note, immune cell numbers within the structurally unaffected *Dsg2*^*MT*^ myocardium did not deviate from those found in wild-type control myocardium (Fig. 4a).

To gain insight into inflammatory activity occurring during scar formation and maturation, we assessed the mRNA expression of pro- and anti-inflammatory cytokines (Fig. 4b). *Tnf*α, *Il1β*, and *Il10* were significantly upregulated at 4 weeks and their expression remained significantly upregulated by 12 weeks albeit at reduced levels. The mRNA expression of galectin 3 (*Lgals3*), which exerts profibrotic effects and is produced by activated macrophages [56], was highly elevated in 4 and 12 week-old *Dsg2*^*MT*^ mice, although expression was substantially lower at 12 weeks.

### Inflammatory cells and cytokine production persist in the chronic disease phase

**Fig. 5:**
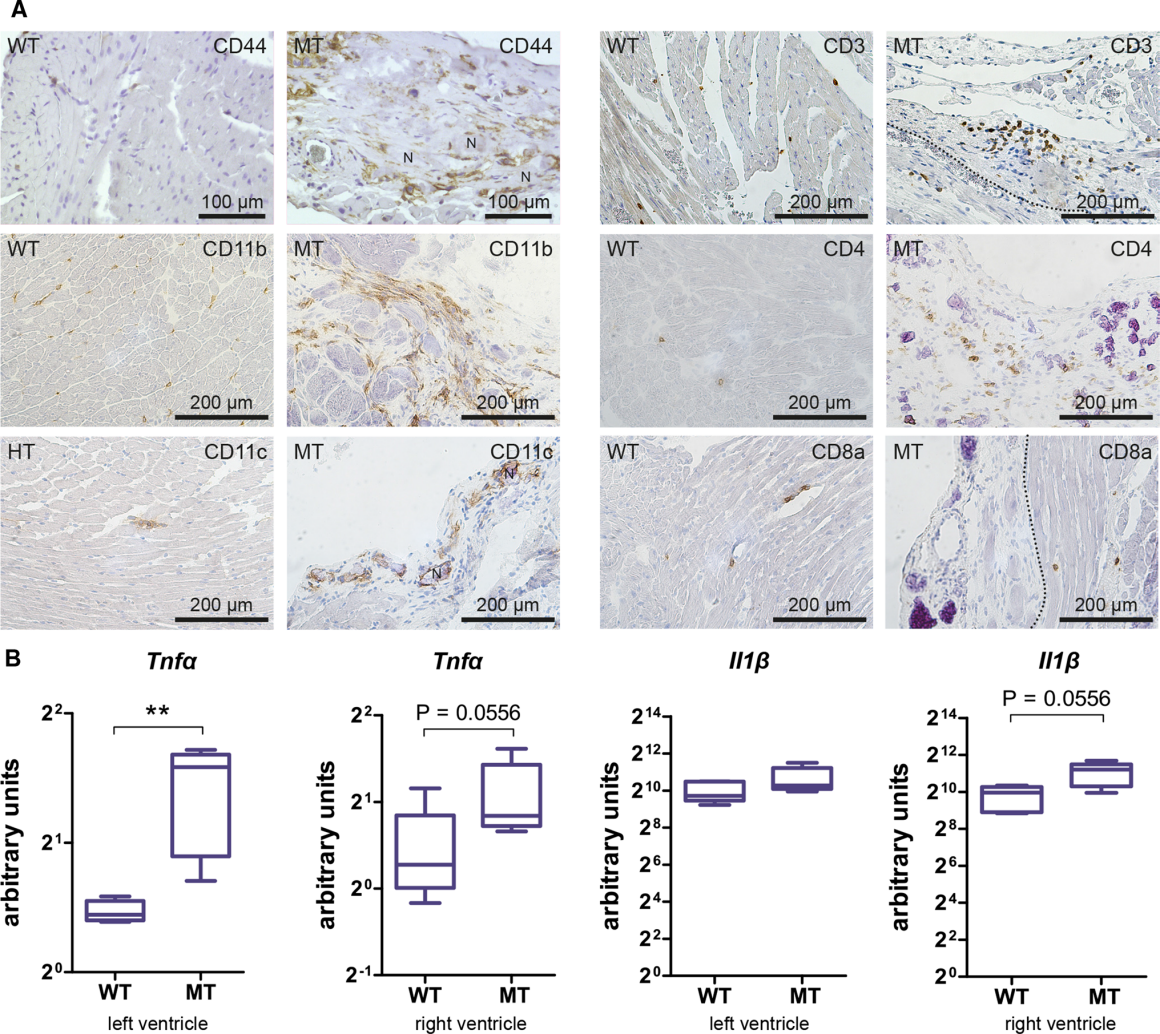
The inflammatory response persists during the chronic disease phase in *Dsg2*^*MT*^ mice. **a** The images depict immunohistochemical stainings detecting distinct immune cells in the myocardium of wild-type (WT) and in fibrotic or calcified necrotic (N) scars of *Dsg2*^*MT*^ (MT) mice at 30–32 weeks (*n* = 4–5 per genotype). Many CD44^+^ immune cells are detected. Note that the majority of the scar-associated immune cells are CD11b^+^ macrophages and CD11c^+^ macrophages/dendritic cells. A cluster of CD3^+^ T cells next to a vein is depicted in the upper right picture. Scattered, spindle-shaped CD4^+^ T cells are detectable in scar tissue whereas CD8a^+^ T cells are only very rarely detected in wild-type and non-lesioned mutant hearts. **b** Tukey's whisker plots show the mRNA expression in arbitrary units (ratio of target and housekeeping gene Hmbs) of the inflammatory cytokines *Tnfα* and *Il1β* in the right and left ventricles of 30–32 week-old *Dsg2*^*MT*^ (MT) and wild-type control mice (WT; *n* = 4–5 per genotype). Mann Whitney *U* test was applied (*p < 0.05 and **p < 0.01)

During the chronic phase of AC fibrotic and calcified scars persisted and increasing interstitial fibrosis was observed (Fig. 3g, h). In the absence of active replacement, we observed that an increased number of CD44^+^ immune cells remained in calcified and fibrotic scars (Fig. 5a). They encompassed CD11b^+^ macrophages and CD11c^+^ dendritic cells (Fig. 5a). Of note, macrophages were also elevated in interstitial fibrosis. In addition, increased CD3^+^ T cells were detected in the different lesion types (Fig. 5a). Anti-CD4 staining identified cells, some of which were not round as to be expected of typical T-helper cells but were rather spindle-shaped and probably resemble a subtype of dendritic cells (right panel in Fig. 5a). An increased number of CD8a^+^ cells could not be detected in the lesioned myocardium (lower right of Fig. 5a).

The mRNA expression of the inflammatory cytokine *Tnfα* remained significantly increased in right and left ventricles during chronic disease progression. Additionally, *Il1β* was slightly elevated in the right ventricles (Fig. 5b).

### Longitudinal assessment of immune cells and cytokine expression reveals different activity levels and activation types

**Fig. 6:**
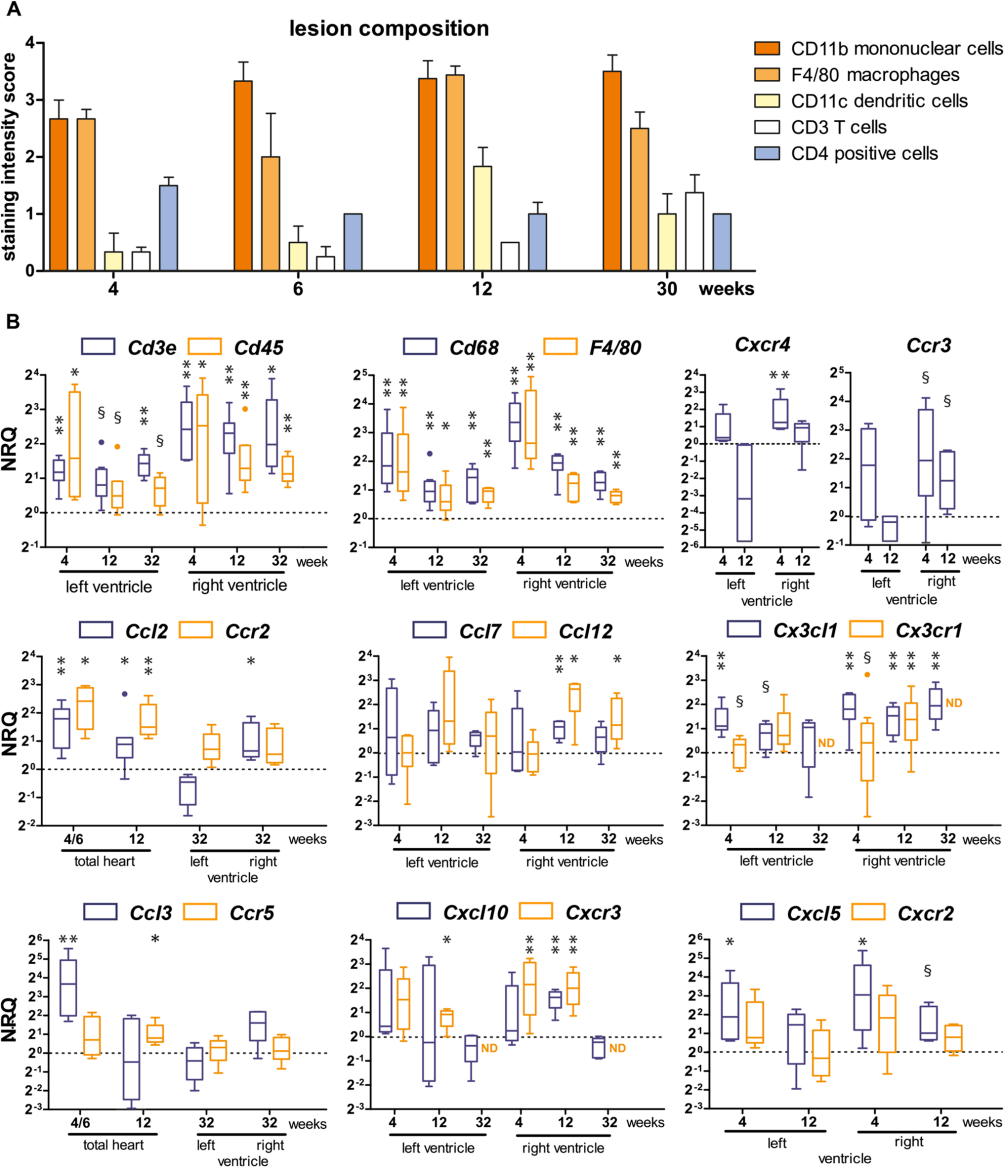
Profiling the immune response from the acute to the chronic stage of cardiomyopathy in *Dsg2*^*MT*^ mice reveals persistent upregulation of macrophages together with dendritic cells and T cells as well as phase-specific chemokine/chemokine receptor upregulation. **a** The histogram depicts the results of a semiquantitative microscopical intensity score assessment of immunohistochemical immune cell detection in myocardial scars of *Dsg2*^*MT*^ mice (*n* = 3–5 for each age group). **b** Tukey's whisker plots show the results of qRT-PCR (normalized relative quantification; NRQ) performed on RNA isolates from right and left ventricles of *Dsg2*^*MT*^ and wild-type controls (dotted lines). Expression was assessed by using non-parametric Mann Whitney tests: *p < 0.05, **p < 0.01; ^§^*p* > 0.05 and < 0.06. For details see Supplementary Table 6 and 7. The increase of *Cd45* (immune cells), *Cd68* and *F4/80* (monocytes/macrophages), and *Cd3e* (T cells) mRNA supports the semiquantitative immunohistochemical results in **a**. Furthermore, the mRNA expression data suggest that the immune response is more pronounced in the right ventricle than in the left ventricle

To semiquantitatively compare the changing levels of phagocytic cells and T cells during tissue remodeling, the staining intensities of antibodies directed against the different cell populations were visually assessed in *Dsg2*^*MT*^ hearts during the acute disease phase at 4, 6 and 12 weeks and the chronic disease phase at 32 weeks (summary in Fig. 6a; examples of immunostaining in Figs. 4a and 5a). As expected, the most intense reactions were observed for the macrophage markers CD11b and F4/80. A somewhat less intense reaction was detected for the dendritic cell marker CD11c, which peaked at 12 weeks. CD3^+^ T cells appeared to increase from 4 to 32 weeks. The population of CD4^+^ cells, which consists of few round-shaped T cells and many spindle-shaped dendritic cells [63], slightly decreased over time.

In support of the immunohistochemical findings, the mRNA expression of the general immune cell marker *Cd45*, the T cell marker *Cd3e,* and the macrophage markers *Cd68* and *F4/80 (Emr1)* were elevated to various degrees in right and left ventricles of different disease stages. The strongest upregulation was noted for the macrophage markers with peak expression preferentially in the right ventricle of 4 week-old *Dsg2*^*MT*^ mice when scars were cell-rich and contained little collagen (Fig. 6b). Furthermore, increased expression of *Cxcr4*, which is expressed on bone marrow-derived immune cells, and of *Ccr3*, which is abundant in Th2 cells, was also detected (Fig. 6b).

Chemokine profiles were examined next since they dictate the composition of recruited blood-borne immune cells and tissue-specific macrophage differentiation [7, 43, 50, 57]. The results highlight the predominant involvement of the right ventricle in the inflammatory response. This is consistent with the increased immune cell marker expression in the right ventricles (top panel of Fig. 6b). In the following, we will therefore solely summarize the observations in the right ventricles or total hearts.

Specifically, *Ccl2* mRNA and its cognate receptor *Ccr2* mRNA, which are expressed in classical inflammatory monocytes, were upregulated during disease onset (Fig. 2a) and remained upregulated in the acute and chronic phases (Fig. 6b). The mouse-specific MCP-1-related CCL12 and CCL7 are other chemokines, which recruit CCR2^+^ cells. *Ccl12* was significantly upregulated but only from the chronic phase transition onwards, and *Ccl7* only at 12 weeks (Fig. 6b). The chemokine CX3CL1 attracts CX3CR1^+^ monocytes, which are involved in tissue repair. The mRNA of both was increased in the acute and chronic phases (for minor changes during disease onset see Fig. 2b).

*Ccl3* and *Ccr5*, which are one of the earliest upregulated ligand-receptor pairs (see Figs. 1d, 2a), were still elevated at 4 weeks but declined thereafter. The mRNA expression of the chemokine *Cxcl10* and its corresponding receptor *Cxcr3*, both of which were increased during disease onset (Fig. 2b), remained elevated at 4 and 12 weeks but returned to wild-type levels during the chronic disease stage.

*Cxcl5*, which encodes a chemokine that recruits CXCR2^+^ neutrophil granulocytes, was upregulated early on during disease initiation (Fig. 2b) but declined during the acute phase.

### Distinct macrophage populations accumulate and differentiate in the hearts of AC mice

**Fig. 7:**
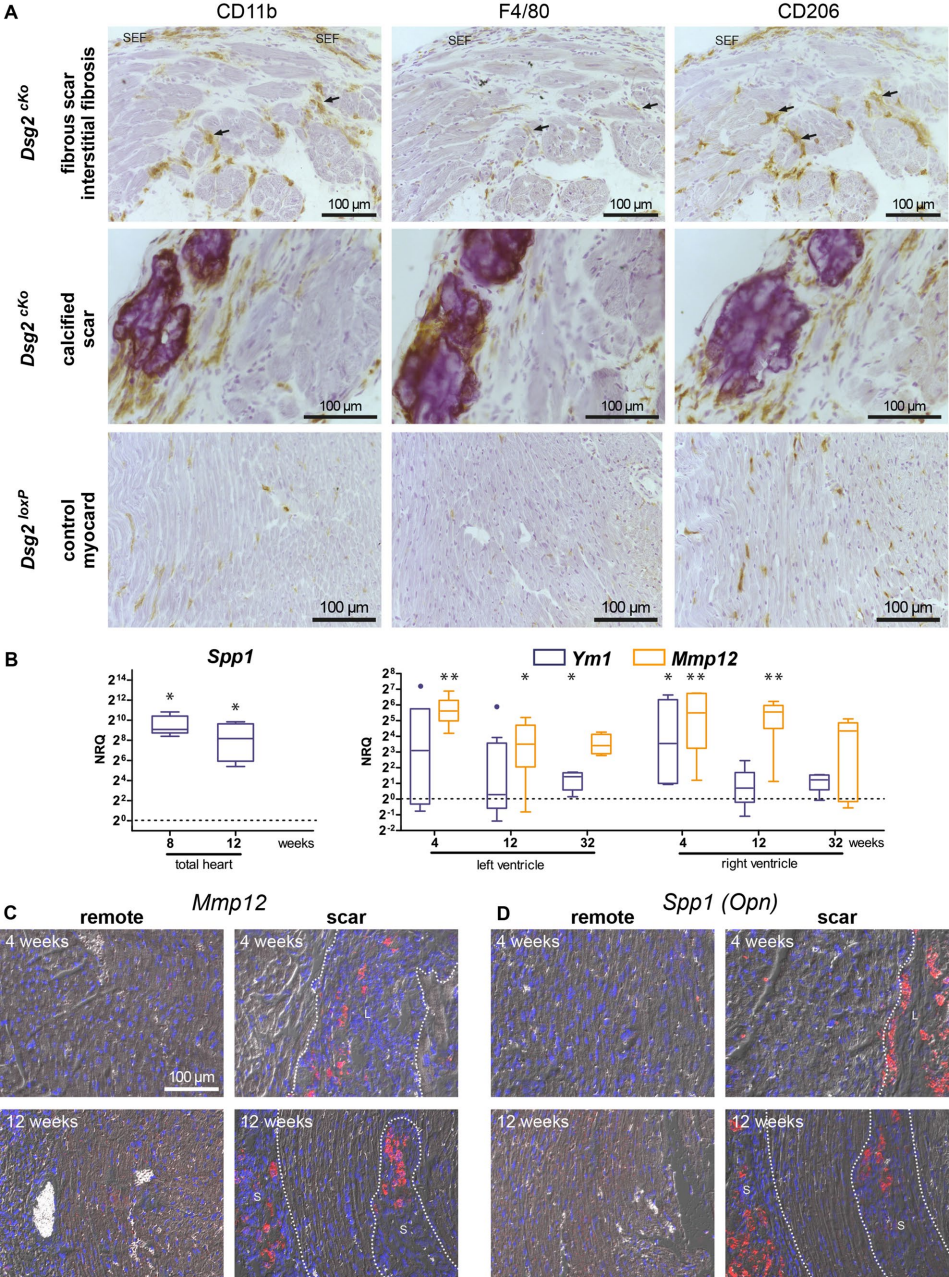
Macrophage distribution and macrophage marker expression is lesion type-specific. **a** depicts the immunolocalization of the macrophage markers CD11b, F4/80 and CD206 in serial cardiac sections (from left to right) of *Dsg2*^*cKO*^ mice with interstitial fibrosis and fibrotic or calcified scars and of *Dsg2*^*loxP*^ control mice at 35 weeks (n = 3 and n = 2, respectively). SEF marks a subepicardial fibrotic scar, arrows point to areas with interstitial fibrosis. **b** Tukey's whisker plots show the quantification (normalized relative quantification; NRQ) of RNAs encoding the macrophage markers *Spp1*, *Ym1,* and *Mmp12* in *Dsg2*^*MT*^ and wild-type control mice (the dotted line indicating relative mean levels of the wildtype). Mann Whitney tests were applied to compare *Dsg2*^*WT*^ and *Dsg2*^*MT*^ mice at each time point: *p < 0.05, **p < 0.01. For more details see Supplementary Table 7. **c** Microscopy of in situ hybridizations detecting *Mmp12* and *Spp1* mRNA (red signal) and nuclei (blue) in paraffin sections of *Dsg2*^*cKO*^ or *Dsg2*^*MT*^ and corresponding control hearts at the age of 4 and 12 weeks. Sections of 3–4 *Dsg2*^*MT/cKO*^ and *2–3 Dsg2*^*WT/loxP*^ hearts were assessed for each age group. Lesions are demarcated by broken lines

Since the microenvironment of a tissue impinges on macrophage differentiation [26, 57] macrophage phenotype and distribution were further characterized. Even healthy myocardium contains a considerable number of homogeneously distributed resident macrophages that express the macrophage-specific CD11b and the CD206 antigen, a marker for anti-inflammatory and reparative macrophages (lower panel of Fig. 7a). F4/80 immunolabelling revealed comparatively weak staining. All three macrophage markers were detected at increased levels in mature scars and interstitial fibrosis of *Dsg2*^*cKO*^ mice (upper panels in Fig. 7a). CD206 and CD11b showed similar staining intensity, whereas the F4/80 staining was again fainter. We then studied the mRNA expression of osteopontin (*Spp1*), which is a marker for reparative macrophages [49, 55, 57], the matrix metalloproteinase 12, a macrophage-specific elastase expressed in pro-inflammatory macrophages [4], and the lectin YM1 (CHI3L3), which is an indicator for reparative or alternatively activated macrophages [45]. *Spp1* mRNA stayed elevated and continued to be significantly increased in 8 and 12 week-old *Dsg2*^*MT*^ mice (Fig. 7b). *Mmp12* mRNA expression was elevated in the left and right ventricles throughout the acute and chronic disease phase. *Ym1* mRNA expression was significantly elevated during the early acute phase and decreased toward the chronic stage.

To examine the impact of the tissue microenvironment [26], we studied *Mmp12* and *Spp1* mRNA expression by in situ hybridization (Fig. 7c). *Mmp12* and *Spp1* mRNA-expressing macrophage-like cells were confined to scar tissue in *Dsg2*^*MT*^ and *Dsg2*^*cKO*^ hearts. Since the remote and healthy myocardium were negative, despite the presence of macrophages, we concluded that *Mmp12*- and *Spp1*-expressing macrophages are recruited to and/or differentiate exclusively in scar tissue.

## Discussion

### Disease stage-specific inflammatory responses occur in *Dsg2*^*cKO*^ and *Dsg2*^*MT*^ mice

The data presented in this paper provide overwhelming evidence for a contribution of inflammatory cells to all stages of murine AC, specifically:

- *Disease onset:* We find that immune cells are even detectable in lesions that can only be identified microscopically. Necrotic cardiomyocytes are surrounded by CD44^+^ inflammatory cells. MPO^+^ neutrophil granulocytes were shown to be a major immune cell type, which is in line with previous observations of hematoxylin–eosin-stained sections in another *Dsg2*-related murine AC model [53]. The increase in *Cxcl5*/*Cxcr2* mRNA expression also testifies to neutrophil granulocyte recruitment. A major function of these cells is most likely the removal of dying cardiomyocytes.
- *Acute disease progression:* This disease stage is the most dynamic. It is characterized by the changing morphology of maturing scars with concomitant changes in immune cell infiltrates and chemokine signaling. Neutrophil granulocytes are gradually replaced by macrophages. This involves recruitment and differentiation of macrophages. Remarkably, *Spp1* and *Mmp12* are exclusively produced in macrophages that are localized in scar tissue. The recruitment and activation of different immune cell types are reflected in the upregulation of *Ccl2*-*Ccl7*-*Ccl12*/*Ccr2*, *Ccl3*/*Ccr5,* and *Cx3cl1*/*Cx3cr1* and *Cxcl10*/*Cxcr3* expression. *Tnfα* and *Il1β* upregulation are further signs of increased inflammation. Both cytokines are known to be produced primarily by inflammatory monocytes and macrophages [48]. On the other hand, tissue remodeling by fibrotic replacement and increased fibrosis are evidenced by an increase in *Lgals3* mRNA and anti-Tenascin C staining. In parallel to the non-specific immune response, a limited adaptive immune cell response kicks in, which is reflected by a minor increase in CD45R^+^ B cells and considerable upregulation of CD3^+^ T cells including CD4^+^ but not CD8a^+^ cells.
- *Chronic disease progression:* Ongoing interstitial fibrosis and increasing cardiodilation are the main morphologic features of this stage. We now provide substantial evidence that inflammation persists albeit at a reduced level in comparison to the acute phase. Thus, the classical inflammation marker *Tnfα* remains increased and a slight elevation of *Il1β* is still detectable. Furthermore, distinct macrophage populations are distinguishably attesting to the ongoing inflammatory remodeling. T cells persist in the chronic disease phase and likely modulate disease progression.

The immune response reported for another *Dsg2* knockout model [14, 15] differs considerably from our observations. The most likely explanation for the difference is that the onset of the disease occurs only when mice reach adulthood, i.e. after 8 weeks, and appears to be less severe in that genetic constellation.

### Early stages of arrhythmogenic cardiomyopathy share features with ischemic myocardial infarction

When looking at the sequence of tissue alterations and inflammation, similarities to myocardial infarction are readily apparent, which have been characterized in much detail using different murine ischemia models [43, 54]. The pathogenesis is triggered by cardiomyocyte necrosis followed by inflammation, scar formation and maturation, and variable long-term responses. The inflammatory reaction sets in within a few hours of ischemia. Neutrophil granulocytes together with inflammatory Ly6C^hi^ M1 monocytes and T cells are the first to invade the ischemic regions. At the same time, multiple chemokines are upregulated including *Ccl2*, *Ccl3*, *Ccl7* and *Cxcl10* [3], which are either slightly or significantly increased at disease onset in *Dsg2* mutants. Our analyses of AC mice furthermore revealed that the corresponding chemokine receptors *Ccr2*, *Ccr5,* and *Cxcr3* are also elevated. Of particular note, the initial increase in *Ccl3*/*Ccr5* and *Il10* that we have noted at AC disease onset has also been observed in myocardial infarction [23]. It was shown to be due to the recruitment of regulatory CD4^+^ T cells, which limit the inflammatory response. These T_reg_ cells are attracted by CCL3/CCR5 interaction and produce IL10 [21].

The next phase of post-ischemia starts within 4 days and lasts 2–4 weeks. It is characterized by changes in the inflammatory cell repertoire [54]. Neutrophil granulocytes die by apoptosis while an increasing number of Ly6C^lo^, CX3CR1^+^, non-classical monocytes is recruited to the lesion. Increased *Ym1*, *Cd206*, *Il10* and *Spp1* mRNA indicate that lesion-associated monocytes differentiate into M2 macrophages [57]. The increased expression of *Ym1*, *Il 10*, *Spp1,* and *Cx3cl1*/*Cx3cr1* in the AC model at 4 weeks suggest that comparable inflammatory mechanisms are at work. The change in immune cell composition is flanked by collagen production by myofibroblasts in the myocardial infarct model [16]. Similar phenomena are observed during the acute phase of Dsg2-related AC. Thus, the pro-inflammatory cytokines *Tnfα* and *Il1β* were detected together with the mRNA of the pro-fibrotic cytokines *Lgals3* and, as previously described, also *Tgfβ1* and *Tgfβ3* [38]. T cells persist in the myocardial infarction and AC models. Of particular interest are beside CD4^+^ T_reg_ cells and CD4^−^/CD8^−^ T cells, which are involved in organ repair [21].

Subsequent scar maturation is reflected by collagen fiber bundling and reorganization accompanied by a reduction in immune cells and chemokine production in the myocardial infarction model [3, 16, 57]. We find, in contrast, a persistent enrichment of macrophages and T cells and upregulation of mRNAs coding for the chemokines and chemokine receptors *Ccl2*, *Ccl7*/*Ccr2* and *Cxcl10*/*Cxcr3*. In parallel, pathologic hypertrophy occurs in the chronic disease phase [31]. Together, these findings point to fundamental differences between lesions formed in myocardial infarct and AC.

It has been reported that Ly6C^hi^ monocytes, M1 macrophages, dendritic cells, and T cells re-appear in chronic ischemic heart failure within weeks or even months after infarct healing [54]. Maybe, this situation is comparable to the continuing presence of CD11b^+^ and CD206^+^ macrophages, CD11c^+^ cells, as well as CD3^+^ T cells that we observed in myocardial scars of AC mice at 30–32 weeks. The reduction in *Ym1* and the continuously high *Mmp12* expression supports a shift towards an M1-like phenotype within the fibrosed myocardium. On the other hand, most if not all cardiac macrophages express the M2 phenotype-associated CD206 antigen in *Dsg2*-mutant mice so that the pathophysiological role of macrophages in AC needs further investigation.

### Inflammation impacts on the onset and progression of arrhythmogenic cardiomyopathy

Our observations that lesions consisting of necrotic cardiomyocytes are the first structural indication of disease onset and that an inflammatory, phagocyte-dominated infiltrate appears only next to necrotic cardiomyocyte but not in normal-appearing myocardium are in full agreement with the sequence of events first proposed by Pilichou and coworkers for another murine AC model [53]. Our observations further show, that cardiomyocyte necrosis is triggered during the most intense postnatal maturation and growth phase of the murine heart, i.e. between 2 and 6 weeks [22, 32, 61], and consistently activates a stereotypical inflammatory repair program, which also includes adverse effects on myocardial structure and function. We, therefore, interpret the finding of necrotic cardiomyocytes next to regions, where the cellular debris had already been cleared by neutrophils and inflammatory macrophages, as a consequence of the damaging effects of effector proteases and reactive oxygen species released by inflammatory cells. The chronic nature of this response is reflected by the continued presence of macrophages, dendritic cells, and T cells in scar tissue. Furthermore, the previously reported connexin 43 redistribution in cardiomyocytes next to immune cell-enriched scars, alterations of actin distribution and actin isoform expression as well as pathological cardiomyocyte hypertrophy in murine *Dsg2* mutants [31, 38, 39] are known to be induced by the macrophage-derived cytokines TNFα, IL1β, TGFβ and also CX3CL1 [24, 27, 28, 36, 47, 66]. These cytokines are also likely involved in the formation of interstitial fibrosis that spreads from the established scars and may thereby contribute to the formation of arrhythmogenic substrates [27]. Finally, the replacement and functional modulation of resident cardiac macrophages, which are known to support electrical excitation propagation [35], by infiltrating macrophages may further contribute to the arrhythmogenic phenotype. The almost inescapable conclusion from all these observations together with the reports on other murine models [15, 51, 53] and human AC patients [5, 8, 11, 46] is that inflammation is a major "driver" of AC progression.

### What are the implications of our findings for human AC patients?

Using murine AC models allows temporal and spatial resolution of disease pathogenesis that cannot be achieved in human patients. Topologically-restricted and continuously recurring lesions are further obstacles in tracking histopathological alterations in human AC patients. The murine AC models are particularly useful to dissect early disease stages, which are most relevant for pediatric AC patients [46, 62]. Their clinical phenotype is especially severe and the observed lesions have been compared to those formed in myocardial infarction [9]. In adult AC patients inflammatory immune cell infiltrates consisting either of neutrophils, macrophages, T lymphocytes, and mast cells or primarily of lymphocytes are detected in severely affected hearts [8, 12]. Similar to our AC mouse models infiltrates are confined to areas of extensive fibro-fatty replacement and necrotic cardiomyocytes.

## Electronic supplementary material

Below is the link to the electronic supplementary material.

Supplementary file1 (PDF 526 kb)

## Data Availability

Primary data will be made available upon request.
