# Supplementary material for: Inflammation shapes pathogenesis of murine arrhythmogenic cardiomyopathy
Source: Basic Res Cardiol. 2020 Jun 12;115(4):42. doi: 10.1007/s00395-020-0803-5 (PMC7289786; doi:10.1007/s00395-020-0803-5)
Supplement: Supplementary file 1 — Supplementary file1 (PDF 526 kb) [file 395_2020_803_MOESM1_ESM.pdf]

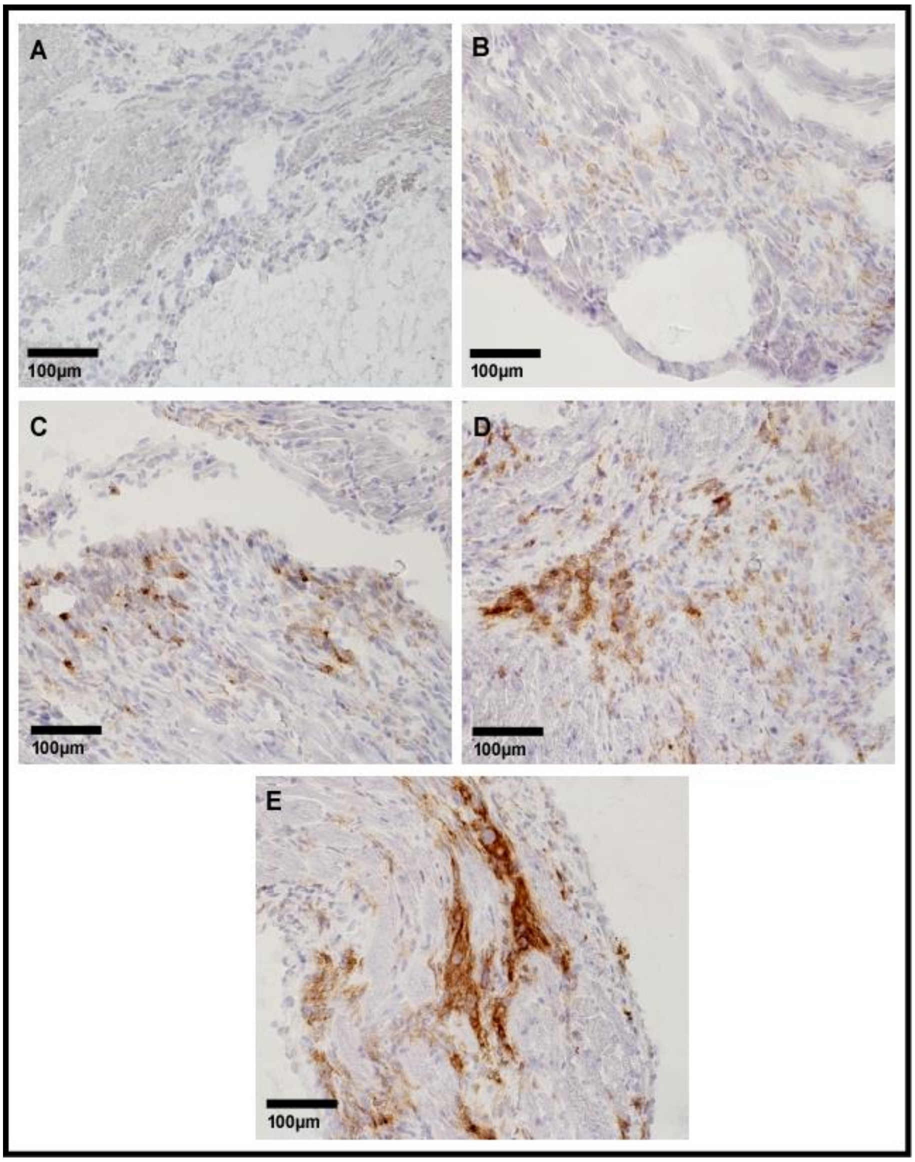

Supplementary Fig. 1 The immunohistochemical images illustrate the semiquantitative score of immunoreactions. The micrographs show CD11b immunostaining in scar tissue of 4-12 week-old *Dsg2*^{MT} hearts. The score was assigned on a scale from 0 to 4: (A) 0 = no or few weakly stained cells; (B) 1 = some scattered and weakly stained cells; (C) 2 = few scattered and strongly stained cells; (D) 3 = many strongly stained cells; and (E) 4 = many tightly packed and strongly stained cells. The figure is taken from the MD thesis of Svenja van der Gaag (2017).

Supplementary Table 1. Ventricle-specific cytokine, chemokine and chemokine receptor mRNA expression in *Dsg2*^{cKO} mice at 18-19 days prior to overt lesion formation (see corresponding Fig. 1)

	Left ventricle			Right ventricle		
	Dsg2^{loxP} n = 4-5	***Dsg2***^{cKO} n = 4-5	**P**	***Dsg2***^{loxP} n = 4	***Dsg2***^{cKO} n = 4-5	**P**
Ccl2	1.00 ± 0.29	3.03 ± 2.56	0.0543	1.00 ± 0.55	41.71 ± 86.01	0.0635
Ccr2	1.00 ± 0.45	1.91 ± 1.15	0.1143	1.33 ± 1.39 $^{\$}$	3.60 ± 2.83	0.3929
Ccl3	1.00 ± 0.50	2.30 ± 1.98	0.4762	1.00 ± 0.52	22.42 ± 30.57	0.0159
Ccr5	1.00 ± 0.28	1.56 ± 1.49	0.8548	0.99 ± 0.31	3.65 ± 2.26	0.0317
Tnfα	1.00 ± 0.16	1.54 ± 0.95	0.1905	1.00 ± 0.51	8.82 ± 12.9	0.2701
Il 1β	1.00 ± 0.59	2.67 ± 2.17	0.1111	1.00 ± 0.51	25.44 ± 47.8	0.0714
Il10	0.99 ± 0.84	1.04 ± 0.47	0.6631	1.00 ± 0,37	5.83 ± 3.78	0.0159

NRQs are given as mean ± standard deviation. The Mann Whitney U test was applied to compare mRNA expression between *Dsg2*^{cKO} and *Dsg2*^{loxP} control mice. * p <0.05; ** p <0.01. $^{\$}$ n =3, because one sample generated no RT-PCR signal.

Supplementary Table 2. Cardiac cytokine, chemokine and chemokine receptor mRNA expression in 2 week-old *Dsg2*^{MT} mice at inflammation onset (see corresponding Fig. 2A)

Total heart				
	Dsg2^{WT} n = 3	***Dsg2***^{MT} n = 6	***Dsg2***^{MT} **Ph+** n = 5	**P**
Ccl2$^{\&}$	1 ± 0.50	2.27 ± 2.49	21.27 ± 18.91^{2*}	0.0381
Ccr2$^{\&}$	1 ± 0.31	1.26 ± 0.58	22.45 ± 23.8^{2*}	0.0103
Ccl3$^{\&}$	1 ± 0.02	2.70 ± 1.23	124 ±129^{2**}	0,0034
Ccr5$^{\&}$	1 ± 0.1	1.63 ± 0.63	14.79 ± 9.62^{2*}	0,0098
	Dsg2^{WT} n = 3	***Dsg2***^{MT} n = 3	***Dsg2***^{MT} **Ph+** n = 4	**P**
Spp1$^{\$}$	0 ± 0.001	0.013 ± 0.015	3.38 ± 3.62^{4*}	0.0139

$^{\&}$Values are given as mean ± standard deviation as NRQ. The Kruskal Wallis test with selected Dunn's Multiple comparison tests as post hoc test were applied.
[1] *Dsg2*^{WT} versus *Dsg2*^{MT}
[2] *Dsg2*^{WT} versus *Dsg2*^{MT} Ph+
* p < 0.05; ** p < 0.01

$^{\$}$Values are given as mean ± standard deviation as *Spp1/Hmbs* ratio. The Kruskal Wallis test with selected Dunn`s Multiple comparison tests as post hoc test were applied.
[3] *Dsg2*^{WT} versus *Dsg2*^{MT}
[4] *Dsg2*^{WT} versus *Dsg2*^{MT} Ph+
* p < 0.05

Supplementary Table 3. Left ventricular chemokine and chemokine receptor mRNA expression in 18-19 days-old *Dsg2*^{cKO} mice at the onset of inflammation (see corresponding Fig. 2 B)

	Left ventricle			
	Dsg2^{loxP}<br>n = 9	***Dsg2***^{cKO}<br>n = 9	***Dsg2***^{cKO} **Ph+**<br>n = 4	**P**
Ccl7	1.00 ± 0.78	7.20 ± 18.28	22.9 ± 17.53	0.0773
Ccl12	0.99 ± 0.33	2.29 ± 3.20	4.22 ± 2.92	0.2445
Cxcl5	0.99 ± 0.58	20.95 ± 52.74	31.94 ± 27.42§**	0.0105
Cxcr2	0.99 ± 2.112	4.36 ± 6.53	4.07 ± 4.29	0.0808
Cxcl10	1.00 ± 0.19	2.72 ± 2.24	11.84 ± 11.92§**	0.0105
Cxcr3	1.00 ± 1.095	2.04 ± 2.84	3.23 ± 1.59	0.0928
Cx3cl1	0.99 ± 0.34	1.00 ± 0.39	1.64 ± 0.38§*	0.0473
Cx3cr1^{X}	0.99 ± 0.29	0.76 ± 0.24	1.43 ± 0.16	0.0358
Cxcr4	1.00 ± 0.37	0.94 ± 0.37	2.04 ± 0.72§*	0.0202
Ccr3	0.99 ± 0.74	5.52 ± 7.73$^{\&*}$	3.13 ± 3.05	0.0396

NRQs are given as mean ± standard deviation. The Kruskal Wallis test with selected Dunn's Multiple comparison tests as post hoc tests were applied.

$^{\&}$ *Dsg2*^{loxP} versus *Dsg2*^{cKO}

§ *Dsg2*^{loxP} versus *Dsg2*^{cKO} Ph+

* $p < 0.05$; ** $p < 0.01$

^{X} *Dsg2*^{loxP} (n = 4); *Dsg2*^{cKO} (n = 3); *Dsg2*^{cKO} Ph+ (n = 4)

Supplementary Table 4. Right ventricular chemokine and chemokine receptor mRNA expression in 18-19 days-old *Dsg2*^{cKO} mice at the onset of inflammation. (see corresponding Fig. 2 B)

	Right ventricle			
	Dsg2^{loxP} n = 8	***Dsg2***^{cKO} n = 10	***Dsg2***^{cKO} **Ph+** n = 4	**P**
Ccl7	1.00 ± 0.59	48.88 ± 133.7	25.66 ± 31.32§*	0.0565
Ccl12	1.00 ± 0.89	2.51 ± 5.34	1.82 ± 1.75	0.6426
Cxcl5	1.00 ± 0.81	52.63 ± 144.7	23.81 ± 26.98§*	0.0465
Cxcr2	1.00 ± 1.01	32.78 ± 79.66	31.28 ± 33.26§*	0.0520
Cxcl10	0.99 ± 0.45	11.4 ± 27.07	14.94 ± 16.74	0.1551
Cxcr3	1.00 ± 0.39	2.16 ± 2.24	13.33 ± 15.83§*	0.0250
Cx3cl1	1.00 ± 0.54	1.21 ± 0.54	1.79 ± 1.16	0.3083
Cx3cr1	1.00 ± 0.60	1.03 ± 0.78	3.93 ± 3.70	0.0474
Cxcr4	1.00 ± 0.93	1.11 ± 0.77	2.38 ± 1.68	0.2224
Ccr3	1.00 ± 0.42	10.43 ± 13.72	27.59 ± 22.18§**	0.0048

NRQs are given as mean ± standard deviation. The Kruskal Wallis test with selected Dunn's Multiple comparison tests as post hoc tests were applied.

$^{\&}$ *Dsg2*^{loxP} versus *Dsg2*^{cKO}

§ *Dsg2*^{loxP} versus *Dsg2*^{cKO} Ph+

* p < 0.05; ** p < 0.01

Supplementary Table 5. Inflammatory and anti-inflammatory cytokine mRNA expression during the acute disease phase in *Dsg2*^{MT} mice (see corresponding Fig. 4)

		Total heart		
		Dsg2^{WT}	***Dsg2***^{MT}	**P**
Tnfα	4 weeks	0.69 ± 0.33	2.38 ± 1.77	0.026
	12 weeks	0.77 ± 0.14	1.24 ± 0.49	0.0348
*Il 1*β	4 weeks	0.32 ± 0.07	3.28 ± 2.88	0.0022
	12 weeks	0.43 ± 0.25	1.15 ± 0.50	0.0070
Il10	4 weeks	2.91 ± 2.31	11.84 ± 6.61	0.0043
	12 weeks	0.31 ± 0.13	0.44 ± 0.145	0.0407
Lgals3	4 weeks	0.22 ± 0.004	2.91 ± 3.18	0.0037
	12 weeks	0.16 ± 0.07	1.71 ± 0.66	0.0021

Values are given as mean ± standard deviation in arbitrary units (target / housekeeping gene ratio). The Mann Whitney U test was applied to compare mRNA expression between wildtype and mutants at each time point in total ventricular samples. 4 week-old mice: n = 6 and 12 week-old mice: n = 7.

Supplementary Table 6: Chemokine and chemokine receptor mRNA expression during the acute and chronic disease phase in left and right ventricles of *Dsg2*^{MT} mice (see corresponding Fig. 6).

		Left ventricle		Right ventricle	
		Dsg2^{WT}	***Dsg2***^{MT}	***Dsg2***^{WT}	***Dsg2***^{MT}
Ccl7	4 weeks	0.41 ± 0.15	1.23 ± 1.32	0.29 ± 0.12	0.57 ± 0.60
	11-14 weeks	0.60 ± 0.20	1.21 ± 0.86	0.15 ± 0.06	0.30 ± 0.07**
	32 weeks	513.4 ± 249	789 ± 194	472 ± 190.1	736 ± 298
Ccl12	4 weeks	0.18 ± 0.09	0.19 ± 0.1	0.14 ± 0.08	0.14 ± 0.07
	11-14 weeks	0.07 ± 0.11	0.36 ± 0.42	0.021 ± 0.008	0.116 ± 0.052*
	32 weeks	43.15 ± 23.9	78.6 ± 73.1	27.0 ± 9.1	79.5 ± 48.4*
Cx3cl1	4 weeks	1.4 ± 0.38	3.71 ± 1.69**	1.17 ± 0.47	4.28 ± 1.91**
	11-14 weeks	1.92 ± 0.29	3.27 ± 1.22§	2.10 ± 0.58	5.72 ± 2.33**
	32 weeks	1046 ±367	1480 ± 588	958 ± 220	2536 ± 882**
Cxcl5	4 weeks	0.046 ± 0.048	0.318 ± 0.344*	0.067 ± 0.038	0.912 ± 1.038*
	11-14 weeks	0.049 ± 0.027	0.119 ± 0.089	0.054 ± 0.035	0.172 ± 0.113$^{\&}$
Cxcl10	4 weeks	0.18 ± 0.11	0.67 ± 0.83	0.128 ±0.14	0. 298 ± 0.283
	11-14 weeks	0.236 ± 0.368	0.694 ± 1.09	0.076 ± 0.016	0.228 ± 0.064**
	32 weeks	123.2 ± 37.45	93.84 ± 37.7	142 ± 68.46	109 ± 30.23
Cx3cr1	4 weeks	0.09 ± 0.04	0.20 ± 0.14§	0.11 ± 0.05	0.31 ± 0.22§
	11-14 weeks	0.018 ± 0.006	0.029 ± 0.017	0.011 ± 0.005	0.048 ± 0.024**
Cxcr2^{1}	4 weeks	2.92 ± 1.94	10.36 ± 10.3	5.47 ± 2.78	24.97 ± 22.95*
	11-14 weeks	3.27 ± 2.58	4.01 ± 3.91	9.17 ± 11.78	16.86 ± 7.67
Cxcr3^{1}	4 weeks	21.28 ± 8.36	70.41 ± 53.66	21.89 ± 12.68	107.7 ± 71.1**
	11-14 weeks	18.10 ± 8.87	31.76 ± 8.30*	19.86 ± 7.57	85.32 ± 41.36**
Cxcr4	4 weeks	0.26 ± 0.09	0.53 ± 0.41*	0.181 ± 0.041	0.677 ± 0.52**
	11-14 weeks	0.26 ± 0.21	0.11 ± 0.13	0.11 ± 0.054	0.186 ± 0.088
Ccl2	32 weeks	455 ± 307	943 ± 326	415 ± 123	877 ± 421*
Ccl3	32 weeks	307 ± 278	242 ± 143	167.5 ± 70.3	513.5 ± 266$^{\&}$
Ccr2	32 weeks	2.93 ± 0.91	5.26 ± 2.11$^{\&}$	1.5 ± 0.62	2.78 ± 1,27$^{\$}$
Ccr5	32 weeks	60.2 ± 40.6	71.8 ± 30.3	64.2 ± 39.2	80.1 ± 34.9

Values are given as mean ± standard deviation in arbitrary units (target / housekeeping gene ratio). The Mann Whitney U test was applied to compare mRNA expression between wildtype and mutants at each time point in total ventricular samples. * p <0.05; ** p <0.01, § p = 0.0519, $^{\&}$ p = 0.0556; $^{\$}$ p = 0.0952. The mRNA expression was assessed in left and right ventricular samples of 5 *Dsg2*^{WT} and 6 *Dsg2*^{MT} mice aged 4 weeks, in 4-6 *Dsg2*^{WT} and 5-6 *Dsg2*^{MT} mice aged 11-14 weeks and in 5 *Dsg2*^{WT} and 4-5 *Dsg2*^{MT} mice aged 29-35 weeks.

[1] Obtained values were multiplied with 10 000.

Supplementary Table 7. Immune cell marker mRNA expression during the acute and chronic disease phase in left and right ventricles of *Dsg2*^{MT} mice (see corresponding Figs. 6 and 7)

		Left ventricle		Right ventricle	
		Dsg2^{WT}	***Dsg2***^{MT}	***Dsg2***^{WT}	***Dsg2***^{MT}
Cd45	4 weeks	0.48 ± 0.22	2.64 ± 2.56*	0.61 ± 0.68	3.63 ± 3.46*
	11-14 weeks	1.93 ± 0.82	3.31 ± 1.74$^{\&}$	0.73 ± 0.22	2.38 ± 1.55**
	32 weeks	1.10 ± 0.34	1.75 ± 0.53$^{\&}$	0.36 ± 0.11	0.89 ± 0.25**
Cd68	4 weeks	0.05 ± 0.007	0.29 ± 0.25**	0.57 ± 0.23	2,66.32 ± 3.57**
	11-14 weeks	0.0446 ± 0.006	0.103 ± 0.053**	0.051 ± 0.004	0.19 ± 0.049**
	32 weeks	0.062 ± 0.01	0.153 ± 0.061**	0.064 ± 0.007	0.16 ± 0.04**
F4/80 (Emr1)	4 weeks	1.92 ± 0.57	9.57 ± 9.50**	1.61 ± 0.35	16.10 ± 17.82*
	11-14 weeks	2.36 ± 0.46	4.12 ± 1.70*	0.59 ± 0.12	1.34 ± 0.42**
	32 weeks	1.06 ± 0.25	1.97 ± 0.36**	0.13 ± 0.03	0.28 ± 0.04**
Cd3	4 weeks	1.074 ± 0.322	2.49 ± 0.677**	0.74 ± 0.52	4.39 ± 2.99**
	11-14 weeks	0.35 ± 0.32	0.72 ± 0.34	0.42 ± 0.30	2.089 ± 0.97**
	32 weeks	0.75 ± 0.34	1.99 ± 0.48**	0.38 ± 0.31	2.15 ± 1.97*
Ym1	4 weeks	0.44 ± 0.24	13.66 ± 24.88	0.95 ± 0.73	33.64 ± 41.42*
	11-14 weeks	0.24 ± 0.14	2.41 ± 4.90	0.33 ± 0.31	0.69 ± 0.55$^{\$}$
	32 weeks	0.08 ± 0.04	0.19 ± 0.07*	0.08 ± 0.09	0.18 ± 0.06$^{\$}$
Mmp12	4 weeks	0.011 ± 0.007	0.65 ± 0.40**	0.01 ± 0.004	0.59 ± 0.54**
	11-14 weeks	0.012 ± 0.004	0.176 ± 0.15*	0.006 ± 0.002	0.288 ± 0.16**
	32 weeks	0.007 ± 0.002	0.087 ± 0.040*	0.0015 ± 0.013	0.20 ± 0.187$^{\$}$

Values are given as mean ± standard deviation in arbitrary units (target / housekeeping gene ratio). The Mann Whitney U test was applied to compare mRNA expression between wildtype and mutants at each time point in total ventricular samples. * p <0.05; ** p <0.01, § p = 0.0519, $^{\&}$ p = 0.0556 or 0.0653; $^{\$}$ p = 0.0932 or p = 0.0952. The mRNA expression was assessed in left and right ventricular samples of 5 *Dsg2*^{WT} and 5-6 *Dsg2*^{MT} mice aged 4 weeks, in 5 *Dsg2*^{WT} and 8 *Dsg2*^{MT} mice aged 11-14 weeks and in 4-5 *Dsg2*^{WT} and 5 *Dsg2*^{MT} mice aged 29-35 weeks.

		Total heart		
		Dsg2^{WT}	***Dsg2***^{MT}	**P**
		N = 5-6	N = 6	
Spp1	8 weeks	0. 004 ± 0.005	2.72 ± 2.51	0.0394
	12 weeks	0.014 ± 0.02	5.63 ± 5.39	0.0112

Values are given as mean ± standard deviation in arbitrary units (target / housekeeping gene ratio). The Mann Whitney U test was applied to compare mRNA expression between wildtype and mutants.
